# Revising Host Phenotypes of Sepsis Using Microbiology

**DOI:** 10.3389/fmed.2021.775511

**Published:** 2021-11-05

**Authors:** Huiying Zhao, Jason N. Kennedy, Shu Wang, Emily B. Brant, Gordon R. Bernard, Kimberley DeMerle, Chung-Chou H. Chang, Derek C. Angus, Christopher W. Seymour

**Affiliations:** ^1^Department of Critical Care Medicine, Peking University People's Hospital, Beijing, China; ^2^Department of Critical Care Medicine, University of Pittsburgh, Pittsburgh, PA, United States; ^3^Clinical Research, Investigation, and Systems Modeling of Acute Illness (CRISMA) Center, Pittsburgh, PA, United States; ^4^Department of Biostatistics, University of Florida, Gainesville, FL, United States; ^5^Department of Medicine, Vanderbilt University Medical Center, Nashville, TN, United States; ^6^Department of Biostatistics, University of Pittsburgh, Pittsburgh, PA, United States; ^7^Department of Emergency Medicine, University of Pittsburgh, Pittsburgh, PA, United States

**Keywords:** phenotype, latent class analysis, host, pathogen, sepsis

## Abstract

**Background:** There is wide heterogeneity in sepsis in causative pathogens, host response, organ dysfunction, and outcomes. Clinical and biologic phenotypes of sepsis are proposed, but the role of pathogen data on sepsis classification is unknown.

**Methods:** We conducted a secondary analysis of the Recombinant Human Activated Protein C (rhAPC) Worldwide Evaluation in Severe Sepsis (PROWESS) Study. We used latent class analysis (LCA) to identify sepsis phenotypes using, (i) only clinical variables (“host model”) and, (ii) combining clinical with microbiology variables (e.g., site of infection, culture-derived pathogen type, and anti-microbial resistance characteristics, “host-pathogen model”). We describe clinical characteristics, serum biomarkers, and outcomes of host and host-pathogen models. We tested the treatment effects of rhAPC by phenotype using Kaplan-Meier curves.

**Results:** Among 1,690 subjects with severe sepsis, latent class modeling derived a 4-class host model and a 4-class host-pathogen model. In the host model, alpha type (*N* = 327, 19%) was younger and had less shock; beta type (N=518, 31%) was older with more comorbidities; gamma type (*N* = 532, 32%) had more pulmonary dysfunction; delta type (*N* = 313, 19%) had more liver, renal and hematologic dysfunction and shock. After the addition of microbiologic variables, 772 (46%) patients changed phenotype membership, and the median probability of phenotype membership increased from 0.95 to 0.97 (*P* < 0.01). When microbiology data were added, the contribution of individual variables to phenotypes showed greater change for beta and gamma types. In beta type, the proportion of abdominal infections (from 20 to 40%) increased, while gamma type patients had an increased rate of lung infections (from 50 to 78%) with worsening pulmonary function. Markers of coagulation such as d-dimer and plasminogen activator inhibitor (PAI)-1 were greater in the beta type and lower in the gamma type. The 28 day mortality was significantly different for individual phenotypes in host and host-pathogen models (both *P* < 0.01). The treatment effect of rhAPC obviously changed in gamma type when microbiology data were added (*P*-values of log rank test changed from 0.047 to 0.780).

**Conclusions:** Sepsis host phenotype assignment was significantly modified when microbiology data were added to clinical variables, increasing cluster cohesiveness and homogeneity.

## Introduction

There are more than 49 million worldwide cases of sepsis annually (1). Despite prompt recognition and treatment, sepsis remains a leading cause of mortality (2, 3). Many trials of candidate sepsis treatments failed to find beneficial effects, in part due to the wide heterogeneity in causative pathogens, host response, and patterns of organ dysfunction. A more precise treatment strategy is needed to move beyond a “one-size-fits-all” bundle (4–7).

Recent work proposed clinical and biologic phenotypes of sepsis that may identify groups for targeted treatment and enrichment strategies in clinical trials (8–17). These studies focus mainly on clinical data in the electronic health record (EHR), protein biomarkers, or molecular data. They do not typically incorporate microbiology or pathogen data as these features are (i) difficult to measure and adjudicate, and (ii) not available at the point-of-care. Despite the inclusion of causative pathogen in leading conceptual models of sepsis (18), its role in sepsis classification using machine learning is unknown.

To address this challenge, we performed a secondary analysis of the Recombinant Human Activated Protein C (rhAPC) Worldwide Evaluation in Severe Sepsis (PROWESS) Study, a large multicenter randomized clinical trial of sepsis patients unique for its detailed microbiology data (19). We aim to determine the effect of adding microbiology data to clinical sepsis phenotypes.

## Methods

The project was approved by the University of Pittsburgh institutional review board and conducted under data use agreements (PRO15110441 and PRO17120315). The original study was approved by the institutional review board at each site, and written informed consent was obtained. The informed consent specified that the data collected will be used for further scientific studies in addition to the original clinical trial (19).

### Data and Study Population

We conducted a secondary analysis of the PROWESS study, which enrolled 1,690 patients with severe sepsis at 164 centers in 11 countries from July 1998 to June 2000. Severe sepsis was defined as a known or suspected infection, 3 or more signs of systemic inflammation, and the sepsis-induced dysfunction of at least one organ or system. Patients were enrolled within 24 h after they met the criteria of severe sepsis. Patients were randomly assigned 1:1 to receive drotrecogin alfa or placebo at each center within 24 h of meeting inclusion criteria (19).

### Clinical and Microbiology Variables for Phenotyping

We selected 24 clinical variables prior to randomization and 3 microbiological variables for analysis. We used clinical variables previously mapped to sepsis phenotypes (15). They included demographic variables (e.g., age, sex, Elixhauser comorbidities), vital signs [e.g., heart rate, respiratory rate, Glasgow coma scale (GCS) score, systolic blood pressure (SBP), temperature, and oxygen saturation (SaO_2_)], markers of inflammation [e.g., white blood cell count (WBC), premature neutrophil count (“bands”)], markers of organ dysfunction or injury [e.g., alanine aminotransferase (ALT), aspartate aminotransferase (AST), total bilirubin, blood urea nitrogen (BUN), creatinine, partial pressure of oxygen (PaO_2_), platelets, and prothrombin time]and serum glucose, sodium, hemoglobin, chloride, and albumin.

The microbiological variables in PROWESS included the site of infection (e.g., bloodstream, central nervous system, genitourinary, abdominal, lung, and others), type of pathogen identified from a positive culture (e.g., mixed, fungus, gram negative, gram positive, and organism negative), and drug resistance (one or more drug resistance vs. no drug resistance).

### Biomarkers, Clinical Outcomes, and Treatment Effects

After phenotypes were assigned, we studied 14 serum biomarkers measured at baseline prior to randomization. They included inflammatory biomarkers [e.g., interleukin (IL)-1b, IL-6, IL-8, IL-10, and tumor necrosis factor (TNF)] and coagulation biomarkers [e.g., antithrombin, d-dimer, factor V, plasminogen activator inhibitor (PAI)-1, plasminogen activity, protein C activity, protein S activity, prothrombin fragment 1–2, and thrombin-antithrombin (TAT) complex].

The primary outcome was 28 day mortality. Secondary outcomes were 90 day mortality and 180 day mortality.

### Statistical Methods

To derive phenotypes, we first explored candidate variable distributions, missingness (Supplementary Table 1), and correlation. We applied log transformations to non-normal data. We handled missing data by using multiple imputations by chained equations (MICE) (20). We included all covariates in the imputation procedure, and modeled variables using logistic, linear, multinomial, or ordinal regression, as appropriate. We evaluated distributions of clustering variables before and after imputation (Supplementary Table 2), and correlation of variables using rank order statistics (Supplementary Figure 1).

We used latent class analysis (LCA) to derive host (24 clinical variables) and host-pathogen (24 clinical plus 3 microbiological variables) phenotypes (21). We determined the optimal number of phenotypes using the minimum Bayesian information criteria (BIC), class size, median probabilities of group membership, entropy, and clinical features of groups. For each patient, we used LCA to produce a posterior probability describing the likelihood of the patient belonging to the phenotype, with posterior probability ranges from 0 to 1. Patients were assigned to the phenotype for which they had the highest posterior probability. We estimated models ranging from two to seven phenotypes (Supplementary Table 3). We determined the optimal number of clusters using a combination of criteria, (i) a smaller BIC, (ii) a higher Entropy, (iii) adequate sample size within cluster, (iv) higher median posterior probabilities of group membership, and (v) clinical characteristics of the clusters. We illustrated the host and host-pathogen models in 2 ways: (i) t-distributed stochastic neighbor embedding (t-SNE) plots (which show the frequency and distribution of phenotype members) and (ii) alluvial plots (which show the change of membership between host and host-pathogen models by phenotypes). We compared the contribution of continuous variables to phenotypes in both host and host-pathogen models using the differences in standardized mean value of each variable.

To quantify the change in phenotypes after addition of microbiology, we measured the mean (SD) probabilities of membership for the assigned group(s). We also compared the proportion of patients in each group using chi square tests. We tested for differences in 28, 90 and 180 day mortality between phenotypes using chi square and Kaplan-Meier curves to illustrate differences in 28 day mortality. We tested the treatment effects for rhAPC by phenotype using Kaplan-Meier curves of 28 day mortality. We conducted 2 sensitivity analyses, (i) excluding variables with high missingness (missing >50%: hemoglobin and premature neutrophil count [bands]) and (ii) using a 5-class model as the optimal fit for the LCA. Analyses were performed with Stata 15.1 (StataCorp, College Station, Texas), and R 3.4.1 (depmixS4 package for LCA; Rtsne package for making t-SNE plots; alluvial package for making alluvial plots, Version: 0.1-2. Bojanowski M and Edwards R; 2016. https://github.com/mbojan/alluvial) with a significance threshold of <0.05 in 2-sided tests.

## Results

### Patients

Among 1,690 subjects, the median age was 64 [IQR: 49–74] years old, 964 (57%) patients were male, and median Elixhauser comorbidity index was 1 [IQR: 0–2] (Table 1, Supplementary Table 4). The primary infection site was lung (54%), compared to abdominal (19%) or genitourinary (11%) infections. A mixed pathogen infection (35%) was the most common, compared to gram positive (22%) or gram negative bacteria alone (16%).

**Table 1:** Characteristics of the host model phenotypes.

**Characteristic**	**All patients**	**α-type**	**β-type**	**γ-type**	**δ-type**	*P*-value^*^
No. of patients (%)	1,690	327 (19.4%)	518 (30.7%)	532 (31.5%)	313 (18.5%)	
Age, median [IQR], years	64 [49–74]	44 [34–55]	71 [63–77]	65 [52–74]	59 [46–73]	<0.01
Gender, no. (%)						0.25
Male	964 (57%)	174 (53%)	310 (60%)	307 (58%)	173 (55%)	
Female	726 (43%)	153 (47%)	208 (40%)	225 (42%)	140 (45%)	
Elixhauser Comorbidities, median [IQR]	1 [0–2]	0 [0–1]	2 [1–3]	1 [0–2]	1 [0–2]	<0.01
**Inflammation**					
Premature neutrophil count (bands), median [IQR], %	1.1 [0.5–2.6]	0.8 [0.3–1.4]	1.1 [0.6–2.2]	1.3 [0.5–3.5]	1.2 [0.4–2.7]	<0.01
Temperature, median [IQR], °C	38.6 [37.6–39.3]	39.0 [38.5–39.5]	38.1 [35.9–38.9]	38.7 [37.7–39.4]	38.6 [37.0–39.6]	<0.01
White blood cell count, median [IQR], ×10^9^/L	14 [9–20]	14 [9–18]	15 [10–21]	13 [7–21]	15 [8–22]	<0.01
**Pulmonary**					
Oxygen saturation, median [IQR], %	95 [90–97]	94 [88–97]	96 [92–98]	94 [90–96]	95 [89–98]	<0.01
Partial pressure of oxygen, arterial, median [IQR], mmHg	76 [62–101]	71 [55–92]	84 [65–125]	71 [62–82]	90 [63–132]	<0.01
Respiratory rate, median [IQR], breaths/min	31 [23–40]	32 [24–40]	28 [19–35]	32 [24–40]	32 [24–40]	<0.01
**Cardiovascular or Hemodynamic**					
Heart rate, median [IQR], beats/min	130 [115–147]	133 [120–148]	122 [105–140]	136 [123–150]	133 [115–150]	<0.01
Systolic blood pressure, median [IQR], mmHg	80 [70–95]	90 [80–110]	85 [69–103]	78 [68–86]	77 [65–92]	<0.01
**Renal**					
Blood urea nitrogen, median [IQR], mg/dL	10 [6–15]	5 [4–7]	11 [7–16]	11 [8–16]	14 [10–20]	<0.01
Creatinine, median [IQR], mg/dL	1.5 [1.0–2.3]	0.9 [0.7–1.1]	1.4 [1.0–2.1]	1.8 [1.3–2.5]	2.3 [1.6–3.4]	<0.01
**Hepatic**					
Alanine transaminase, median [IQR], U/L	28 [16–55]	26 [15–43]	20 [13–31]	27.5 [17–50]	130 [50–395]	<0.01
Aspartate transaminase, median [IQR], U/L	43 [24–93]	37 [22–68]	28 [20–43]	47 [28–85]	246 [102–616]	<0.01
Bilirubin, median [IQR], mg/dL	0.7 [0.4–1.3]	0.7 [0.4–1.3]	0.5 [0.3–0.9]	0.8 [0.5–1.5]	1.0 [0.6–2.2]	<0.01
**Hematologic**					
Hemoglobin, median [IQR], g/dL	11 [9–12]	11 [10–12]	10 [9–12]	11 [9–12]	11 [10–12]	0.02
Platelets, median [IQR], ×10^9^/L	168 [105–240]	193 [140–256]	205 [147–290]	135 [90–199]	129 [71–200]	<0.01
Prothrombin time, median [IQR], secs	19 [17–22]	17 [16–19]	18 [16–20]	20 [18–24]	22 [18–30]	<0.01
**Other**					
Albumin, median [IQR], g/dL	2.0 [1.6–2.4]	2.2 [1.7–2.6]	2.0 [1.6–2.5]	1.9 [1.5−2.3]	2.0 [1.5–2.5]	<0.01
Chloride, median [IQR], mEq/L	106 [101–111]	105 [102–110]	106 [100–111]	107 [103–112]	105 [100–111]	<0.01
Glasgow Coma Scale score, median [IQR]	14 [11–15]	15 [12–15]	14 [9–15]	15 [12–15]	14 [10–15]	<0.01
Glucose, median [IQR], mg/dL	146 [115–196]	133 [112–162]	163 [124–227]	144 [112–198.5]	144 [108–196]	<0.01
Sodium, median [IQR], mEp/L	139 [135–143]	139 [135–142]	139 [135–143]	139 [136–142]	139 [135–144]	0.34
**Outcomes**					
28 day mortality, no. (%)	469 (28%)	29 (9%)	157 (30%)	152 (29%)	131 (42%)	<0.01
90 day mortality, no. (%)	593 (35%)	43 (13%)	210 (41%)	188 (35%)	152 (49%)	<0.01
180 day mortality, no. (%)	638 (38%)	51 (16%)	233 (45%)	198 (37%)	156 (50%)	<0.01

* *Kruskal-Wallis used for continuous and or chi-square for categorical comparisons, across four phenotypes. IQR, interquartile range*.

### Host Model

**Figure 1:**
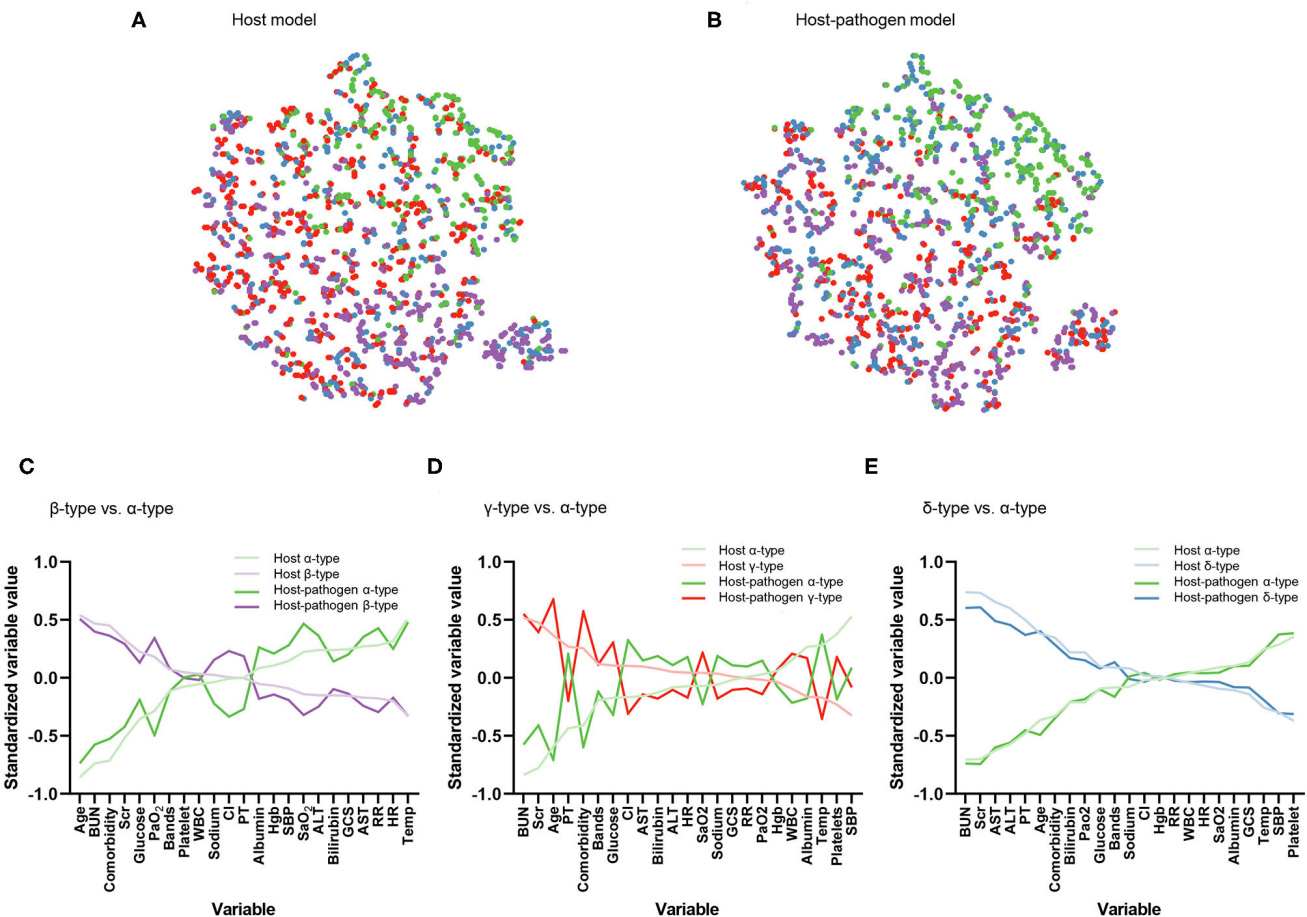
Visualization of phenotype assignments and comparison of clinical variables that contribute to phenotypes. **(A)** Visualization of phenotype assignments in host, **(B)** host-pathogen model using t-distributed stochastic neighbor embedding (t-SNE) plot. Green, purple, red, and blue dots represent α-type, β-type, γ-type and δ-type, respectively. Phenotype members have a similar frequency and distribution across models. **(C)** Differences in standardized mean value of each variable ranked from maximum positive to negative separation (x-axis). Dark lines correspond to host model. Light lines correspond to same comparisons but from host-pathogen model. Plot compares β-type (purple) to α-type (green). Variables ranked on the left x-axis are greater in β-type than α-type (e.g., age, BUN, and comorbidity) while those on the right x-axis are lower in β-type than α-type (e.g., temperature, heart rate). **(D)** Comparison between γ-type (red) and α-type (green). **(E)** Comparison between δ-type (blue) and α-type (green).

Using 24 clinical variables in the latent class analysis (host model), we determined that a 4-class model was the optimal fit [applied labels alpha (α), beta (β), gamma (γ), and delta (δ) types]. Entropy in all models was 0.75 or greater, and the BIC decreased as class number increased from 2 to 4. The median probability of group membership was high (>95%, Supplementary Table 3, Supplementary Figure 2). Phenotypes ranged in size from 19 to 32% of the cohort, and differed broadly in clinical characteristics (Table 1, Figure 1). Consistent with prior data (15), patients with the α-type (19%) were younger and had less shock, β-type (31%) were older and had greater comorbidity, γ-type (32%) had more pulmonary dysfunction, and δ-type (19%) had more liver, renal, and hematologic dysfunction and shock.

### Host Pathogen Model

**Figure 2:**
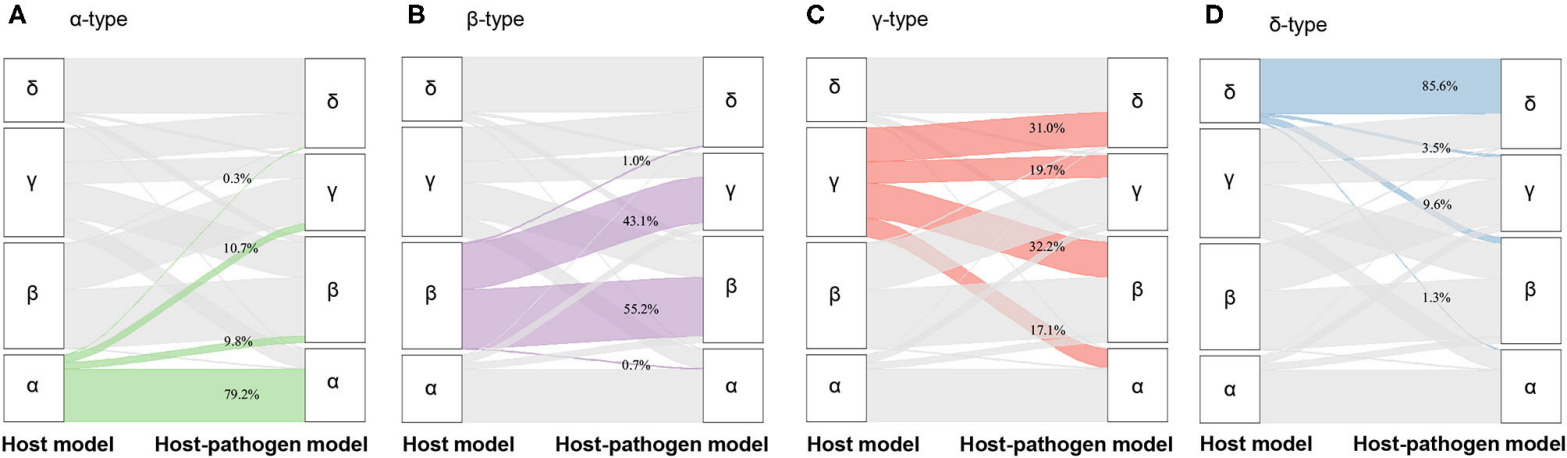
Alluvial plot showing the change of membership from host model to host-pathogen model. **(A)** The change of membership from α-type of host model (green, left column, *N* = 327) to host-pathogen model (right column), **(B)** from β-type of host model (purple, left column, *N* = 518) to host-pathogen model (right column), **(C)** from γ-type of host model (red, left column, *N* = 532) to host-pathogen model (right column), **(D)** from δ-type of host model (blue, left column, *N* = 313) to host-pathogen model (right column).

When 3 microbiological variables were included in the latent class analysis (host-pathogen model), a 4-class model again demonstrated optimal fit (also applied labels α, β, γ, and δ types) (Supplementary Table 3, Supplementary Figure 2). We visualized patients using t-SNE plots (Figures 1A,B) and found that the proportion of phenotype members was similar in host and host-pathogen models. However, 772 of 1,690 (46%) patients changed phenotypes, particularly the β (45%) and γ-types (80%) (Figure 2, Supplementary Table 5). The host-pathogen phenotypes had higher median membership probabilities than host phenotypes alone (host: 0.95 vs. host-pathogen: 0.97, *P* < 0.01, Supplementary Table 5). Among patients who rearranged phenotypes in the host-pathogen model, the initial host model membership probability was lower than patients who did not change (median 0.90 vs. 0.98, *p* < 0.01, Supplementary Table 6).

The contribution of individual variables to phenotypes are ranked before and after including microbiology data. These plots show little change for δ- and α-types, but greater inconsistency for the β- and γ-type variables (Figures 1D–F). For example, among β-type patients, the proportion of abdominal infections (from 20 to 40%) and mixed-type infections (from 36 to 44%) increased, while the proportion of lung infections decreased from 57 to 35%; γ-type patients had an increased rate of lung infections (from 50 to 78%) with worsening pulmonary function (PaO_2_ decreased from 71 to 64 mmHg) (Tables 1, 2, Supplementary Tables 4, 7, 8).

**Table 2:** Example characteristics of β-type and γ-type in host and host-pathogen models.

**Variable**	**Host β-type**	**Host-pathogen β-type**	**Host γ-type**	**Host-pathogen γ-type**
No. of patients (%)	518 (31%)	519 (31%)	532 (32%)	374 (22%)
**Clinical variable**				
Age, median [IQR]	71 [63–77]	69 [58–77]	65 [52–74]	70 [61–77]
Elixhauser comorbidity, median [IQR]	2 [1–3]	1 [1–2]	1 [0–2]	2 [1–3]
Heart rate, beats/min, median [IQR]	122 [105–140]	126 [110–144]	136 [123–150]	127 [112–145]
SBP, mmHg, median [IQR]	85 [69–103]	80 [70–92]	78 [68–86]	83 [69–103]
Bilirubin, mg/dL, median [IQR]	0.5 [0.3–0.9]	0.6 [0.4–1.1]	0.8 [0.5–1.5]	0.6 [0.3–0.9]
Glucose, mg/dL, median [IQR]	163 [124–227]	147 [117–198]	144 [112–199]	166 [127–227]
Oxygen saturation, %, median [IQR]	96 [92–98]	97 [94–98]	94 [90–96]	92 [85–95]
PaO_2_, mmHg, median [IQR]	84 [65–125]	92 [73–138]	71 [62–82]	64 [53–77]
Platelets, × 10^9^/L, median [IQR]	205 [147–290]	175 [116–252]	135 [90–199]	205 [157–281]
Prothrombin time, s, median [IQR]	18 [16–20]	19 [17–23]	20 [18–24]	17 [15–19]
WBC Count, × 10^9^/L, median [IQR]	15 [10–21]	13 [8–19]	13 [7–21]	16 [12–21]
**Microbiological variable**				
**Source**				
Bloodstream, no. (%)	17 (3.3%)	16 (3.1%)	27 (5.1%)	2 (0.5%)
Abdominal, no. (%)	102 (20%)	208 (40%)	120 (23%)	8 (2.1%)
Lung, no. (%)	293 (57%)	183 (35%)	268 (50%)	291 (78%)
**Type**				
Mixed, no. (%)	188 (36%)	230 (44%)	183 (34%)	96 (26%)
Gram positive, no. (%)	87 (17%)	87 (17%)	130 (24%)	67 (18%)
Organism negative, no. (%)	128 (25%)	90 (17%)	109 (21%)	129 (35%)
Drug resistance, no. (%)	133 (32%)	198 (38%)	116 (28%)	64 (17%)

*IQR, interquartile range; PaO_2_, partial pressure of oxygen; SBP, systolic blood pressure; WBC, white blood cell*.

### Correlation With Baseline Biomarkers and 28-Day Mortality

**Figure 3:**
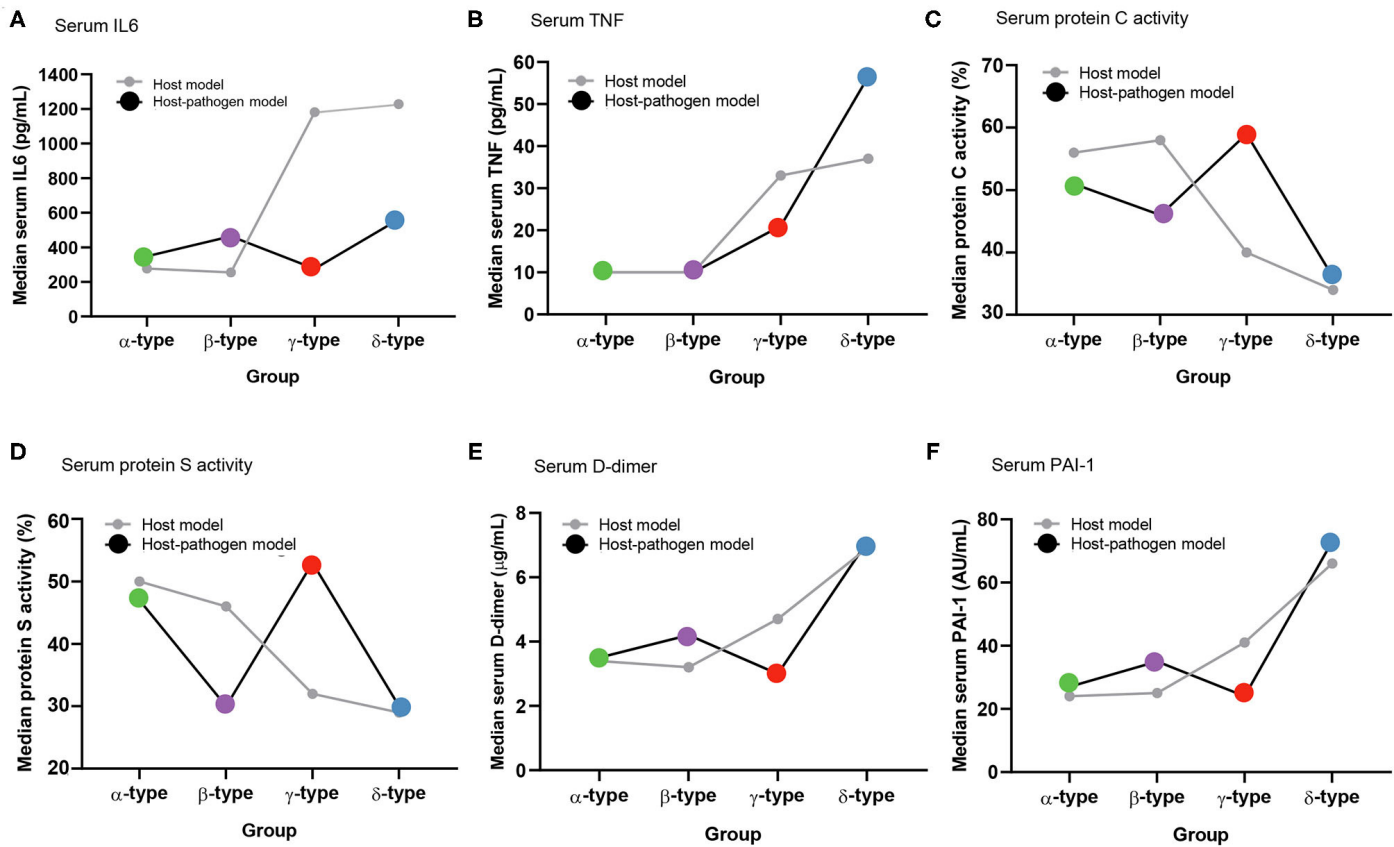
Comparison of median values of biomarkers between host and host-pathogen models across phenotypes. **(A)** Serum IL-6, **(B)** serum TNF-α, **(C)** serum protein C activity, **(D)** serum protein S activity, **(E)** serum D-dimer, and **(F)** serum plasminogen activator inhibitor-1 (PAI-1). Gray lines represent host model, and black lines represent host-pathogen model. Green, purple, red, and blue dots represent α-type, β-type, γ-type and δ-type, respectively.

**Figure 4:**
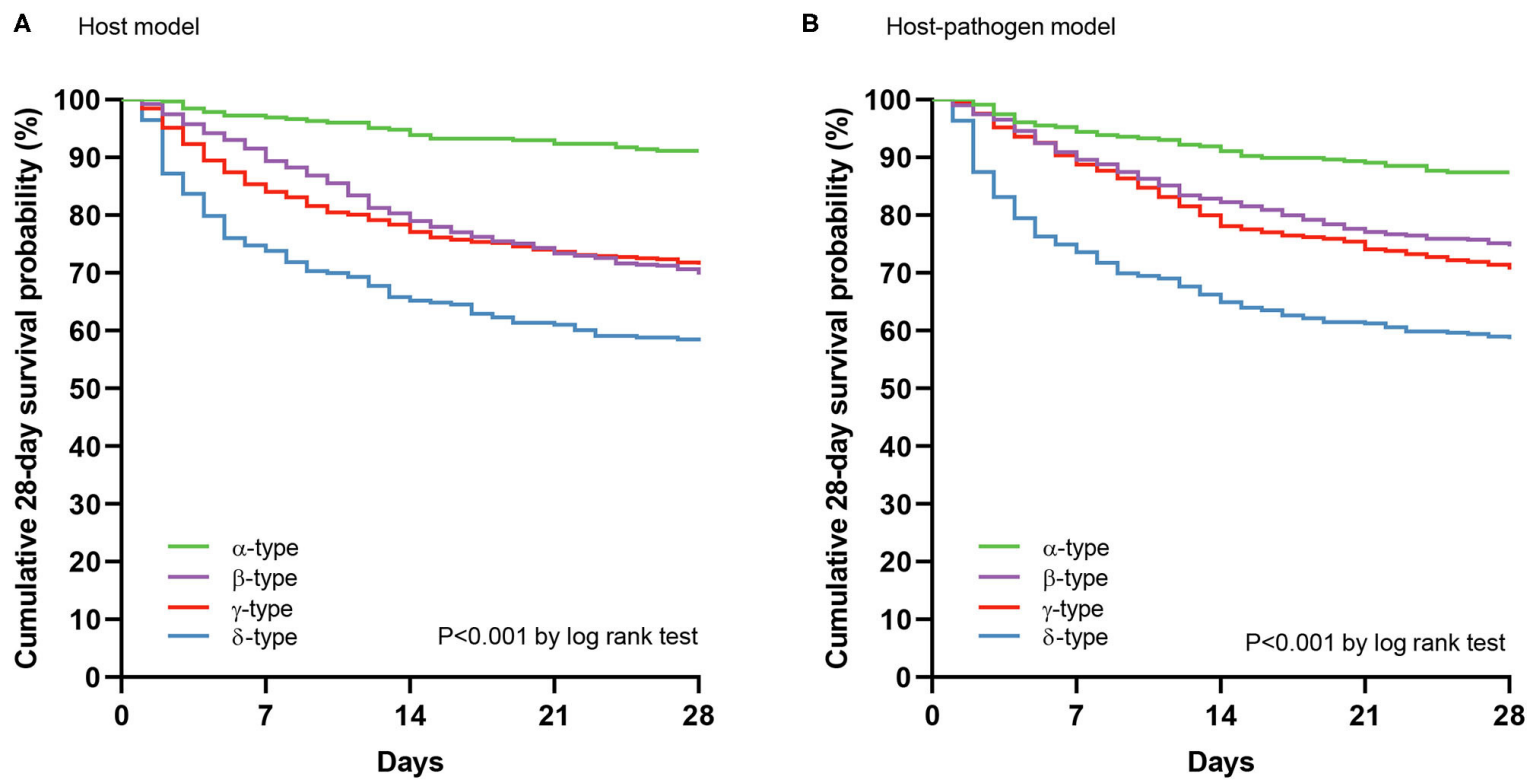
28 day mortality by phenotypes using Kaplan-Meier curves. **(A)** Cumulative survival at 28 days by phenotypes using Kaplan-Meier curves in host model, and **(B)** host-pathogen model. Green, purple, red, and blue lines represent α-type, β-type, γ-type and δ-type, respectively.

Comparing host and host-pathogen models, 13 of 14 biomarkers were significantly different across phenotypes when adding microbiology data (excluding only IL-1b, Supplementary Tables 9, 10). For example, in the β-type, the median level of PAI-1 increased from 25 to 35 AU/mL, and d-dimer increased from 3.2 to 4.2 μg/mL; while PAI-1 (from 41 to 24 AU/mL) and d-dimer (from 4.7 to 3.0 μg/mL) decreased in the γ-type (Figure 3). The cumulative 28 day mortality probability was significantly different for individual phenotypes in host and host-pathogen models (both log-rank *P* < 0.01), but was similar between models. In both models, 90 day and 180 day mortality were also significantly different for individual phenotypes (all chi-square *P* < 0.01), but were similar between models (Figure 4; Table 1, Supplemental Tables 4, 7).

### Treatment Effect for rhAPC by Phenotype After Including Microbiology Variables

**Figure 5:**
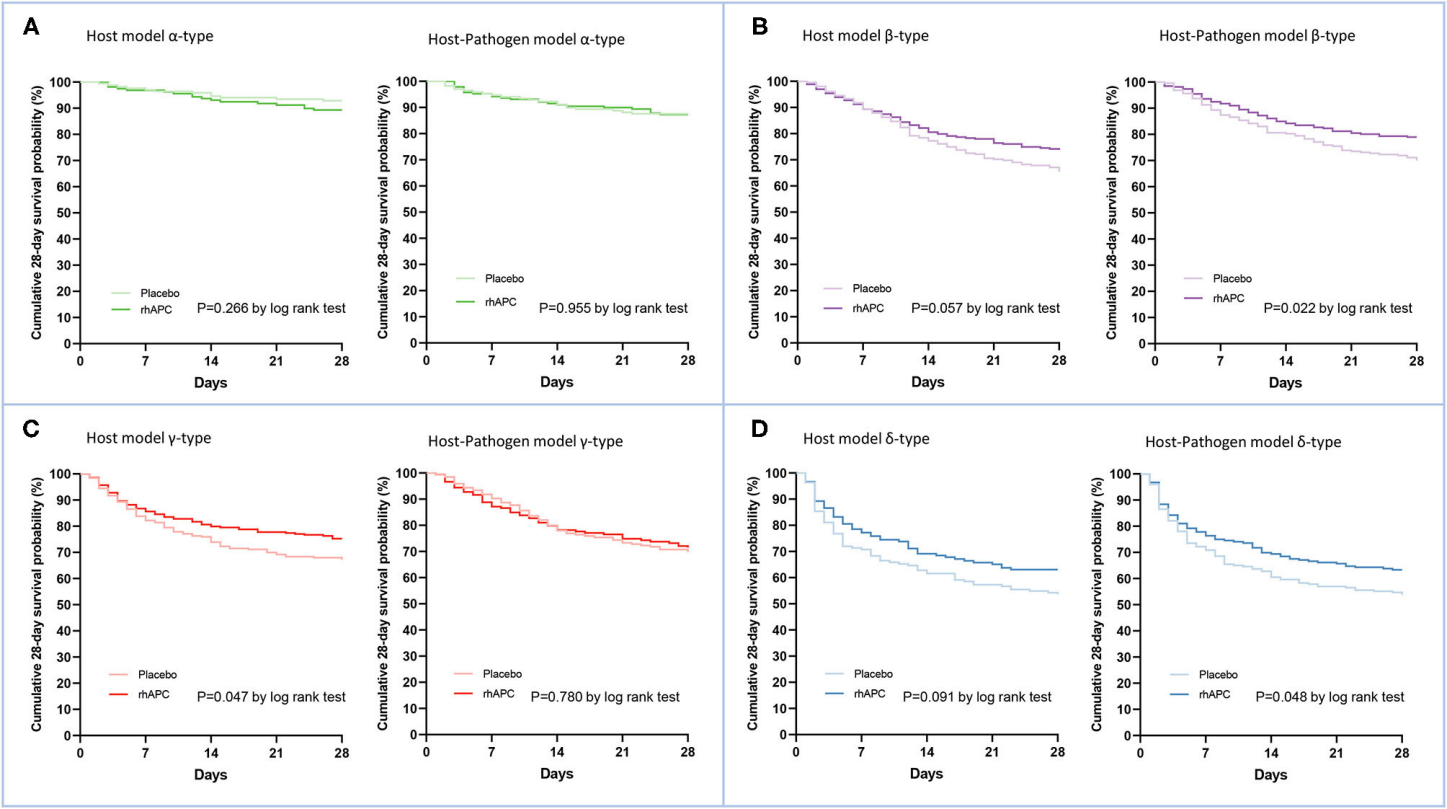
Comparison of the treatments effects for Recombinant Human Activated Protein C by phenotype. **(A)** The comparison of cumulative survival at 28 days using Kaplan-Meier curves between rhAPC group and placebo group in α-type of both host and host-pathogen model, **(B)** β-type, **(C)** γ-type, and **(D)** δ-type. Dark lines correspond to rhAPC group and light lines correspond to placebo group. Green, purple, red, and blue dots represent α-type, β-type, γ-type and δ-type, respectively.

In host model, rhAPC significantly decreased the cumulative 28 day mortality probability in gamma type (*P* = 0.047 by log rank test), while when microbiology variables were added, the 28 day mortality was similar between rhAPC and placebo group (*P* = 0.780 by log rank test) (Figure 5).

### Sensitivity Analysis

To understand the robustness of these results, we derived phenotypes excluding variables with high missingness and found that a 4-class model remained optimal for both host and host-pathogen models (Supplementary Figure 3). In addition, these models had similar frequency and characteristics to phenotypes as the primary analysis (Supplementary Table 11, Supplementary Figure 3). For example, 713 (42%) patients were rearranged when microbiological variables were added, with highest rates of change in the β and γ-type (Supplementary Figure 4). We also explored a 5-class model and found that microbiological variables also rearranged 632 (37%) of patients, increased the probability of membership, and changed variable characteristics in clinically meaningful way (Supplementary Tables 12, 13, Supplementary Figure 5).

## Discussion

In this proof-of-concept analysis, the addition of microbiological variables to host sepsis phenotypes led to meaningful rearrangement of patients, particularly the beta and gamma types. These changes did not modify short or long-term outcomes, but changed the treatment effect for rhAPC in gamma type. This work suggests that pathogen data may have an under-recognized role in sepsis phenotype classification using machine learning methods.

For decades, sepsis has been characterized by the offending pathogen, such as Neisseria meningitis or pneumococcal pneumonia. However, these labels alone do not capture the combined complexity of the host response, tolerance, or damage in sepsis (22). Recent work using machine learning to subtype sepsis did not include pathogen data due to practical measurement challenges during emergency care (8–11, 15, 23). Preliminary work in the PROWESS-SHOCK trial began to use microbiology together with clinical data to propose subphenotypes of septic shock (17). We extend this work by investigating the question, how much does microbiology add beyond that of clinical data alone? This is a key knowledge gap that will guide the embedding of sepsis phenotypes into trials and clinical practice.

We found that the addition of microbiological variables to host phenotypes led to meaningful rearrangement of sepsis patients. A large proportion, particularly of the gamma type, were assigned to a different phenotype. The host pathogen model also appeared to statistically increase in probability of assignment. These changes were not, however, accompanied by changes in patient outcomes by phenotype. We also found that the addition of pathogen data could obviously change the treatment effect for rhAPC in gamma phenotype. It further elaborated the importance of pathogen data to sepsis phenotyping. As a proof of concept analysis, many important steps follow, (i) to reproduce in larger, generalizable cohort; (ii) determine if other treatment effects, perhaps time to antimicrobials or source control, are modified by pathogen informed subtypes.

A challenge to the incorporation of microbiological data into sepsis phenotypes is that these parameters are not routinely available during emergency care or at the time of typical enrollment in clinical trials. Several rapid approaches are under study to identify infection type (e.g., bacterial, viral), or drug resistance. These include multiplex real-time polymerase chain reaction (PCR) systems, next-generation sequencing (NGS) (24–26), and those probing the pathogen specific host response (27, 28). These approaches have complex workflow, a need for rigorous quality control, and a yet-to-be-determined optimal “clinical moment” in bedside care.

This study has several limitations. First, we performed a proof of concept in a single trial with small sample, and generalizability requires further study. Second, the microbiology data were derived from the culture results of the database of PROWESS which could not accurately and completely distinguish the colonization, positive cultured infection, and negative cultured infection. In addition, due to the low incidence, we did not identify multidrug-resistant (MDR) and extensively drug-resistant (XDR) bacteria in the drug resistance variables, these two variables have greater clinical application value. Third, most pathogens were bacteria, with low rates of viral and fungal infection. Additional data is needed to parse through the role of specific viral pathogens to phenotypes. Fourth, missing data were common. Although we used multiple imputation, bias may be introduced for those variables with high missingness. To address this limitation, we excluded variables with high missingness (>50%) in sensitivity analyses and found similar results. Fifth, we compared mortality and treatment effects of rhAPC between host and host-pathogen models using Kaplan-Meier curves which may lead to non-rigorous results. Further need to verify these effects using stratified proportional hazards model in larger sample study. Sixth, the choice of optimal number of clusters is semi-subjective and different statistical approaches are available to determine cluster number. Informed by prior work in SENECA (15), we focused on 4 class models. However, we explored a 5-class model in sensitivity analyses and found similar trends to those observed in the primary analysis.

## Conclusion

Sepsis host phenotype assignment was significantly modified when microbiology data were added to clinical variables, increasing cluster cohesiveness and homogeneity. The clinical significance of these changes and importance for treatment effects in clinical trials remains uncertain.

## Data Availability Statement

The raw data supporting the conclusions of this article will be made available by the authors, without undue reservation.

## Ethics Statement

The studies involving human participants were reviewed and approved by University of Pittsburgh institutional review board. Written informed consent for participation was not required for this study in accordance with the national legislation and the institutional requirements. Written informed consent was not obtained from the individual(s) for the publication of any potentially identifiable images or data included in this article.

## Author Contributions

HZ, JK, DA, and CS contributed to study conception and design. GB, DA, and CS contributed to acquisition of data. HZ, JK, SW, EB, KD, C-CC, DA, and CS contributed to analysis and interpretation of data. HZ and CS drafted the manuscript. CS supervised the study. All authors critically revised the manuscript.

## Funding

HZ was supported in part by grant from Peking University People's Hospital Research and Development Funds (RDY2019-43, derive sepsis phenotypes using electronic medical data and machine learning). CS was supported in part by grants from the National Institutes Health (R35GM119519).

## Conflict of Interest

The authors declare that the research was conducted in the absence of any commercial or financial relationships that could be construed as a potential conflict of interest.

## Publisher's Note

All claims expressed in this article are solely those of the authors and do not necessarily represent those of their affiliated organizations, or those of the publisher, the editors and the reviewers. Any product that may be evaluated in this article, or claim that may be made by its manufacturer, is not guaranteed or endorsed by the publisher.
