# Supplementary material for: Revising Host Phenotypes of Sepsis Using Microbiology
Source: Front Med (Lausanne). 2021 Nov 5;8:775511. doi: 10.3389/fmed.2021.775511 (PMC8602092; doi:10.3389/fmed.2021.775511)
Supplement: Supplementary file 1 [file Data_Sheet_1.docx]

**Supplemental Digital Content**

**Revising Host Phenotypes of Sepsis Using Microbiology**

| **Table of Contents Page** | **Page** |
| --- | --- |
| **Supplemental Figure 1**. Heatmap of correlation between clinical variables for phenotyping | 3 |
| **Supplemental Figure 2**. Bayesian Information Criterion and model entropy by class number from latent class analysis | 4 |
| **Supplemental Figure 3**. Statistical output from latent class analysis in sensitivity analysis excluding variables with high missingness (hemoglobin and bands) | 5 |
| **Supplemental Figure 4.** Alluvial plot showing the change of membership between host and host-pathogen phenotypes in sensitivity analysis excluding variables with high missingness (hemoglobin and bands) | 6 |
| **Supplemental Figure 5.** Alluvial plot showing the change of membership between host and host-pathogen phenotypes in 5-class model | 7 |
| **Supplemental Table 1.** Missing data (N, %) of clinical and microbiological variables | 8 |
| **Supplemental Table 2.** Variables before and after multiple imputation | 10 |
| **Supplemental Table 3**. Statistical output from latent class analysis | 11 |
| **Supplemental Table 4**. Microbiological characteristics and outcomes by host phenotypes | 12 |
| **Supplemental Table** 5. Metrics of model fit between host and clinical and host-pathogen models | 13 |
| **Supplemental Table 6.** Comparison of the probability between membership changed and not changed patients when microbiological variables added | 14 |
| **Supplemental Table 7.** Characteristics of variables by host-pathogen phenotypes | 15 |
| **Supplemental Table 8.** Characteristics of Microbiological variable by phenotypes in host-pathogen model | 17 |
| **Supplemental Table 9.** Biomarkers by host phenotypes | 18 |
| **Supplemental Table 10.** Biomarkers by host-pathogen phenotypes | 19 |
| **Supplemental Table 11.** Statistical output from latent class analysis in sensitivity analysis excluding variables with high missingness (Hemoglobin, premature white cells (%bands)) 46 Revising sepsis phenotypes using microbiology | 20 |
| **Supplemental Table 12.** Metrics of model fit in 5-class model between host and host-pathogen phenotypes | 21 |
| **Supplemental Table 13. Comparison** of microbiological variables in type 2 and type 3 of 5- class model between host and host-pathogen phenotypes | 22 |

**Supplemental Digital Content**

**Supplemental Figure 1. Heatmap of correlation between clinical variables for phenotyping**


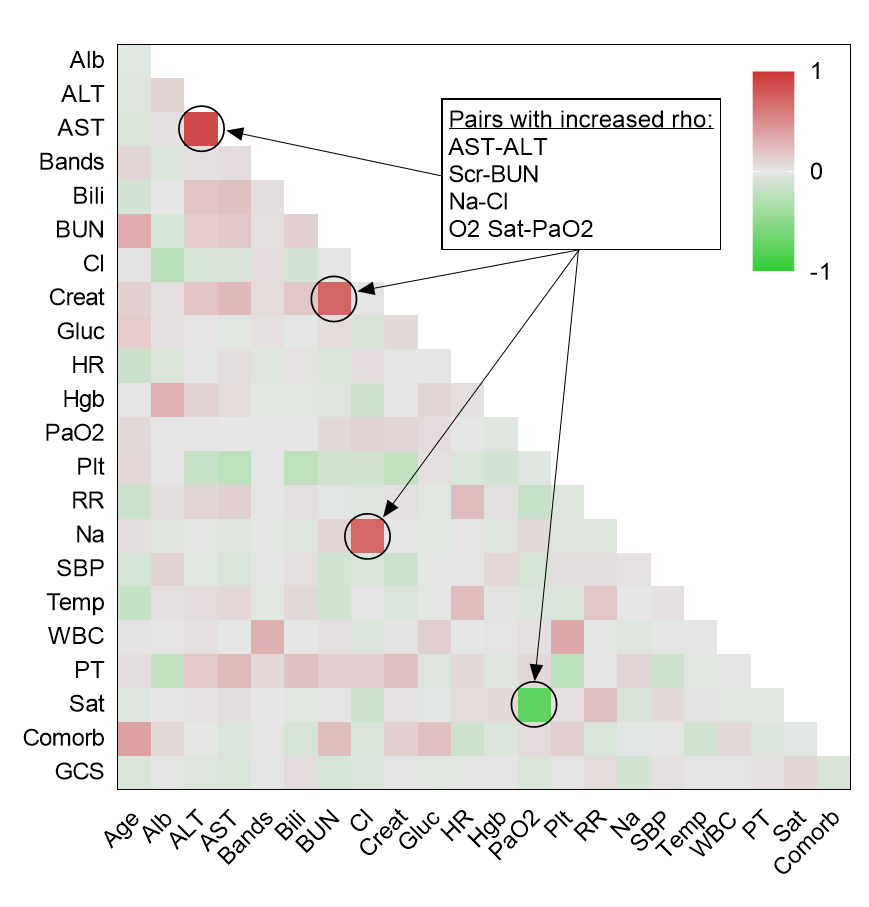


Heatmap shows greater color (red or green) when the Spearman rank order correlation coefficient is greater in positive or negative direction. The call out box identifies pairs of variables with moderate correlation (rho>0.4).

Note: Normalized Sat values were calculated as log(101-Sat), giving a negative relationship between Sat and PaO_2_.

Abbreviations: Alb: albumin; ALT: alanine transaminase; AST: aspartate transaminase; Bili: bilirubin; BUN: blood urea nitrogen; Cl: chloride; Creat: serum creatinine; Gluc: glucose; HR: heart rate; Hgb: hemoglobin; PaO2: arterial oxygen pressure; Plt: platelet count; RR: respiratory rate; SBP: systolic blood pressure; Temp: temperature; WBC: white blood cell count; PT: prothrombin time; Sat: oxygen saturation; Comorb: elixhauser comorbidity; GCS: Glasgow coma scale score.

**Supplemental Figure 2. Bayesian Information Criterion and model entropy by class number from latent class analysis**


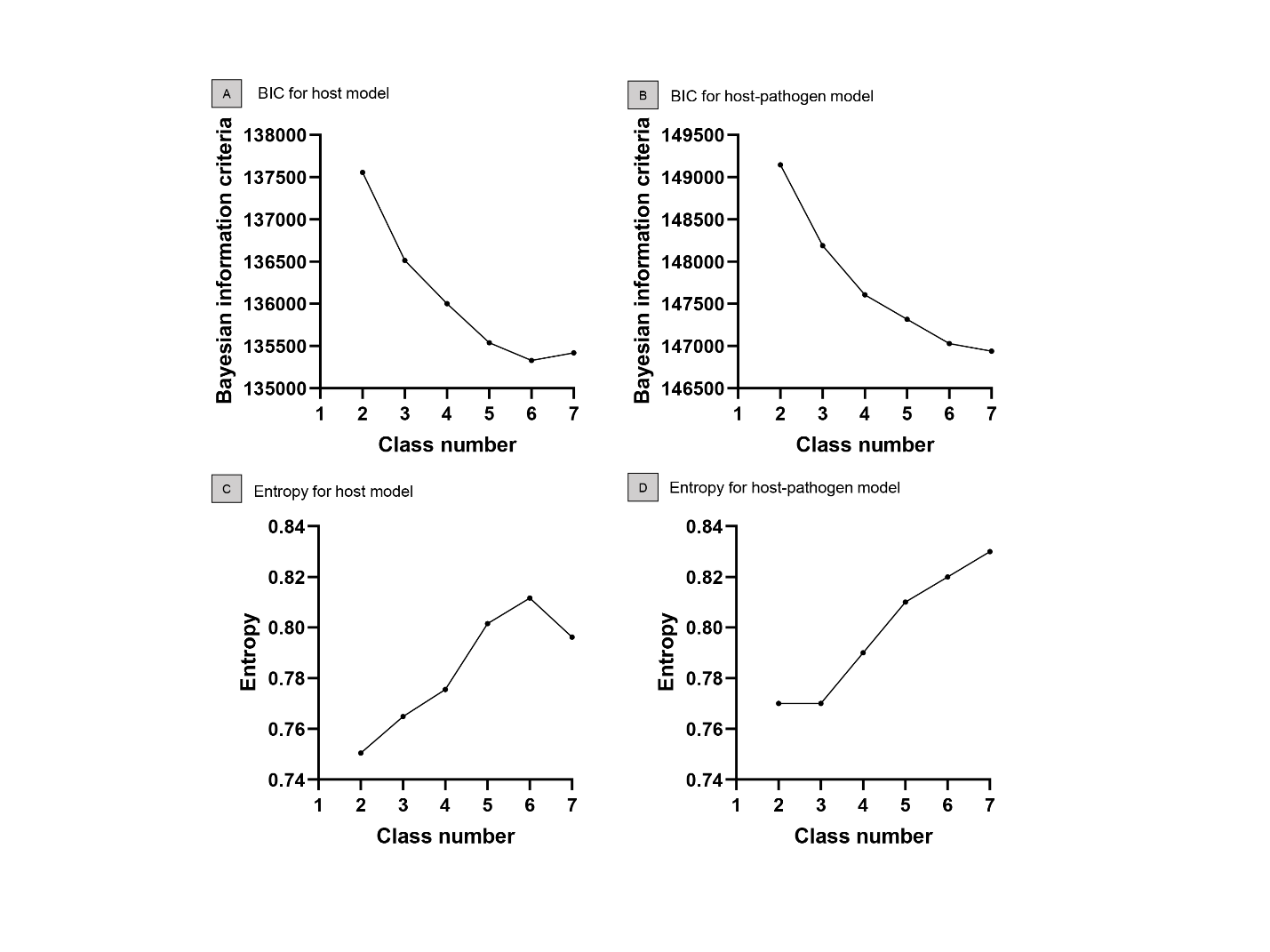


(A) Bayesian information criterion (BIC) by class number in host model, where Y-axis shows the value of BIC and X-axis corresponds to the class number. (B) Bayesian information criterion (BIC) by class number in host-pathogen model, similar plot to panel A. (C) Entropy by class number in host model, where Y-axis shows the value of entropy and X-axis corresponds to the class number. (D) Entropy by class number in host-pathogen model, similar plot to panel C.

**Supplemental Figure 3. Statistical output from latent class analysis in sensitivity analysis excluding variables with high missingness (hemoglobin and bands).**


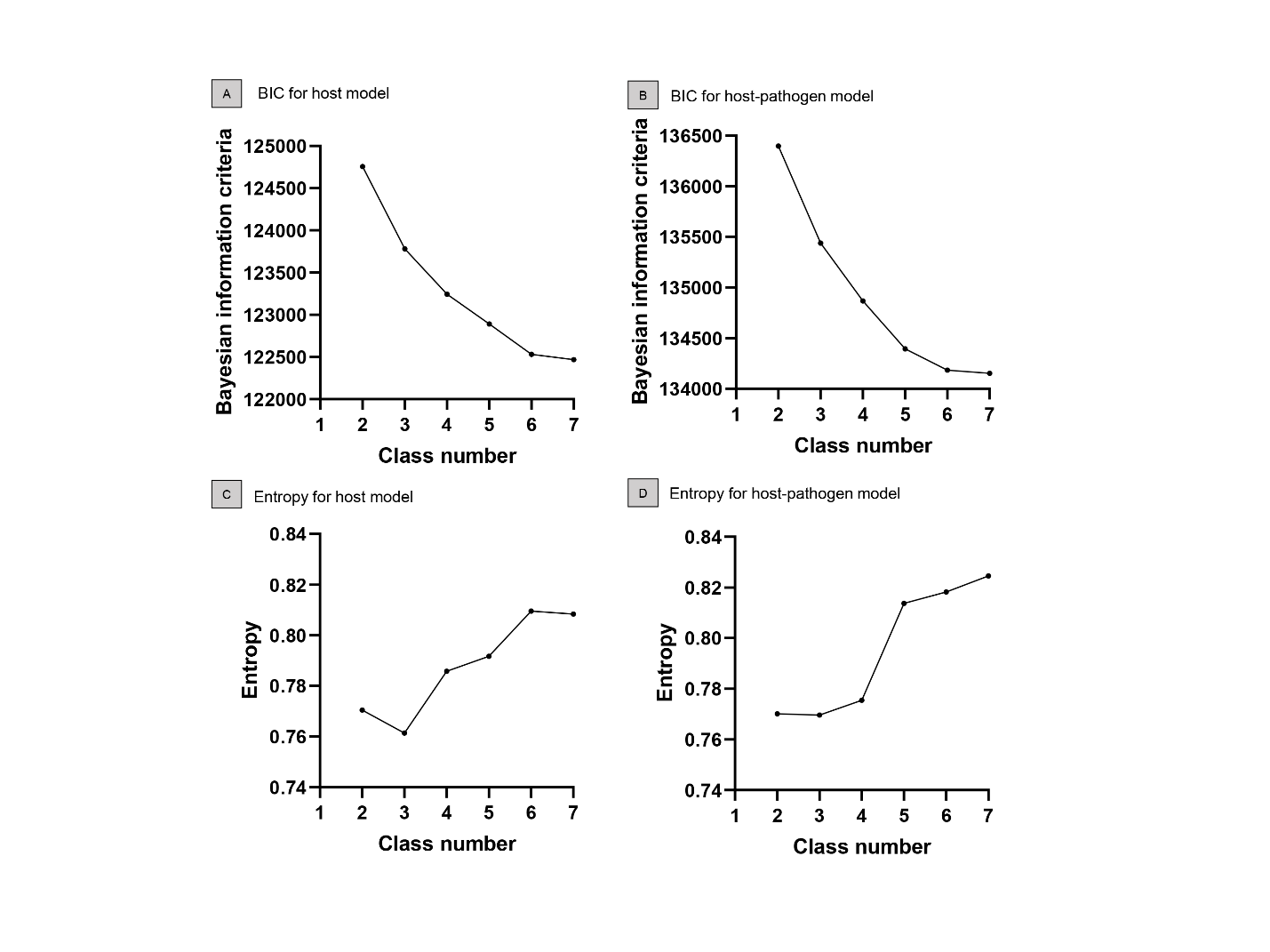


(A) Bayesian information criterion (BIC) by class number in host model, where Y-axis shows the value of BIC and X-axis corresponds to the class number. (B) Bayesian information criterion (BIC) by class number in host-pathogen model, similar plot to panel A. (C) Entropy by class number in host model, where Y-axis shows the value of entropy and X-axis corresponds to the class number. (D) Entropy by class number in host-pathogen model, similar plot to panel C.

**Supplemental Figure 4. Alluvial plot showing the change of membership between host and host-pathogen phenotypes in sensitivity analysis excluding variables with high missingness (hemoglobin and bands).**


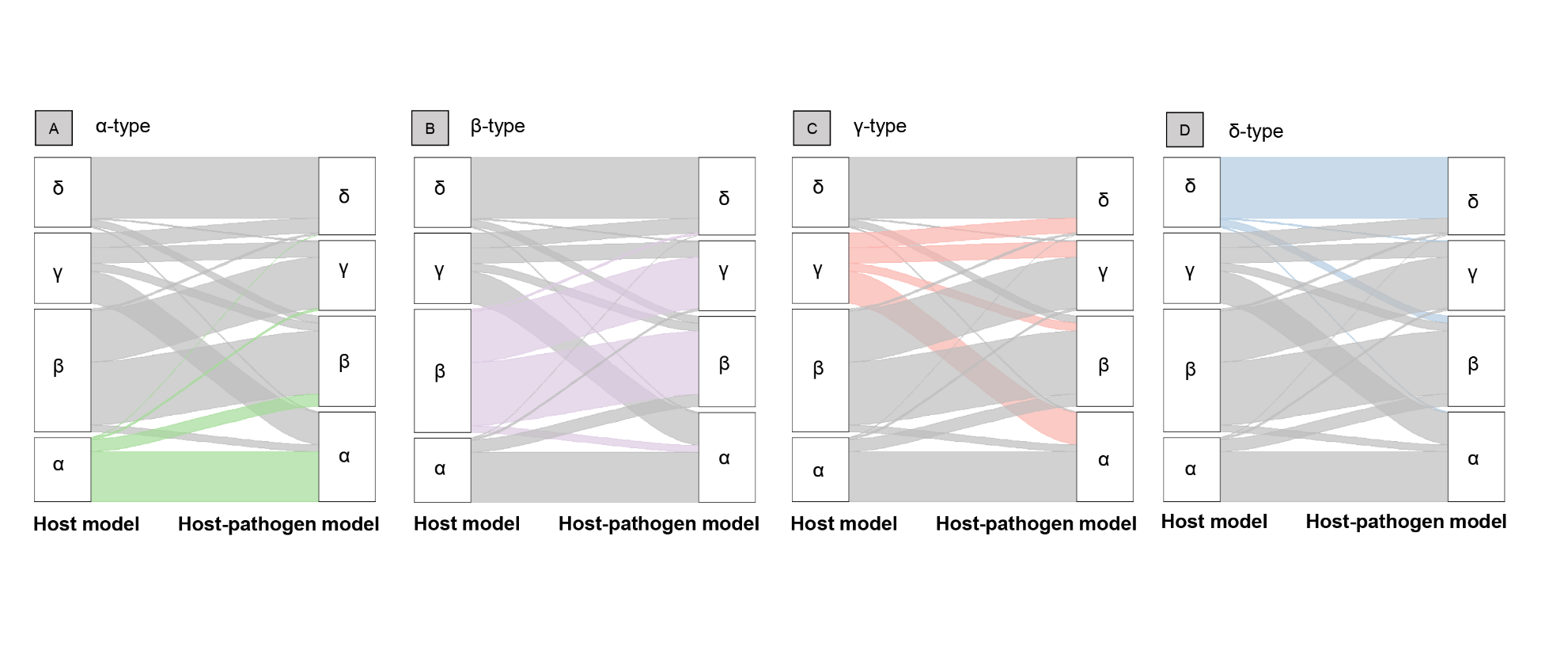
(A) The change of membership from α-type of host model (green, left column, N=331) to host-pathogen model (right column), (B) from β-type of host model (purple, left column, N=635) to host-pathogen model (right column), (C) from γ-type of host model (red, left column, N=362) to host-pathogen model (right column), (D) from δ-type of host model (blue, left column, N=362) to host-pathogen model (right column).

**Supplemental Figure 5. Alluvial plot showing the change of membership between host and host-pathogen phenotypes in 5-class model.**


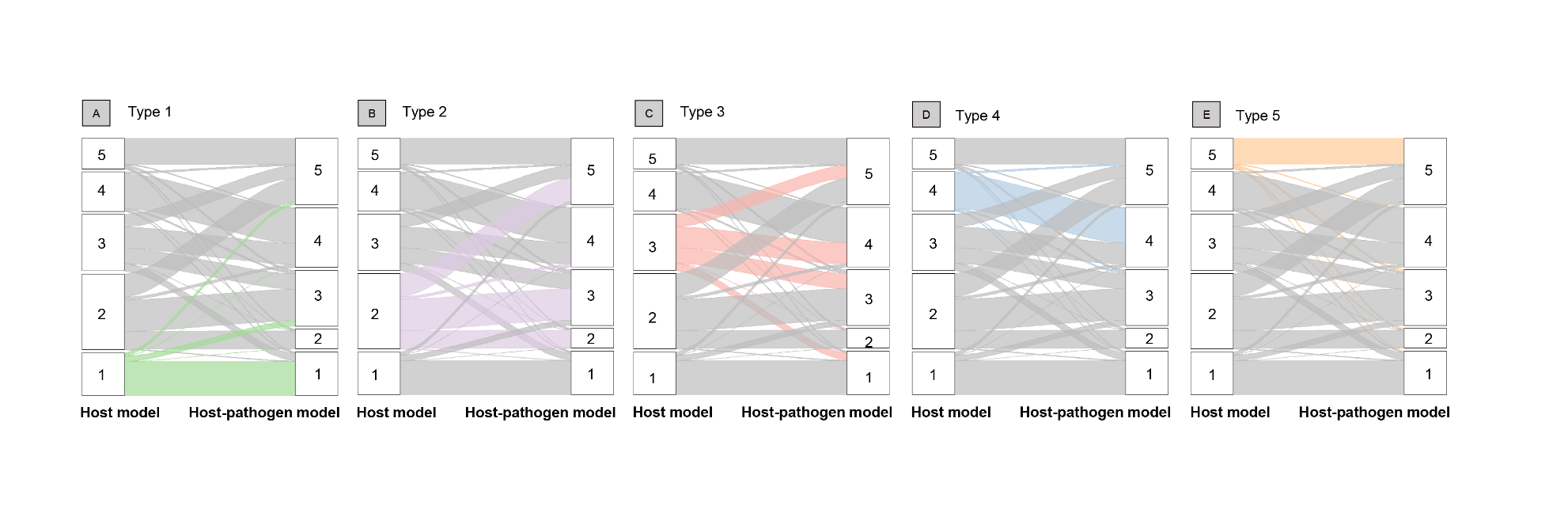


(A) The change of membership from type-1 of host model (green, left column, N=294) to host-pathogen model (right column), (B) from type-2 of host model (purple, left column, N=523) to host-pathogen model (right column), (C) from type-3 of host model (red, left column, N=390) to host-pathogen model (right column), (D) from type-4 of host model (blue, left column, N=272) to host-pathogen model (right column), (F) from type-5 of host model (yellow, left column, N=211) to host-pathogen model (right column).

**Supplemental Table** **1**: **Missing data (N, %) of clinical and microbiological variables**

| **Variable** | | **Missing data** |
| --- | --- | --- |
| **Clinical variables** | |  |
|  | Age | 0 (0%) |
|  | Albumin | 84 (5.0%) |
|  | ALT | 83 (4.9%) |
|  | AST | 83 (4.9%) |
|  | Bands | 1238 (73%) |
|  | Bilirubin | 710 (42%) |
|  | BUN | 43 (2.5%) |
|  | Chloride | 42 (2.5%) |
|  | Creatinine | 4 (0.2%) |
|  | Elixhauser comorbidity | 0 (0%) |
|  | GCS | 181 (11%) |
|  | Gender | 0 (0%) |
|  | Glucose | 103 (6.1%) |
|  | Heart Rate | 0 (0%) |
|  | Hemoglobin | 1033 (61%) |
|  | Oxygen saturation | 239 (14%) |
|  | PaO_2_ | 23 (1.4%) |
|  | Prothrombin time | 132 (7.8%) |
|  | Platelets | 1 (0.1%) |
|  | Respiratory rate | 5 (0.3%) |
|  | Sodium | 0 (0%) |
|  | SBP | 3 (0.2%) |
|  | Temperature | 0 (0%) |
|  | WBC | 0 (0%) |
| **Microbiological variables** | |  |
| Infection site | |  |
|  | Bloodstream, no. (%) | 0 (0%) |
|  | Central nervous system, no. (%) | 0 (0%) |
|  | Genitourinary, no. (%) | 0 (0%) |
|  | Abdominal, no. (%) | 0 (0%) |
|  | Lung, no. (%) | 0 (0%) |
|  | Others, no. (%) | 0 (0%) |
| Type | |  |
|  | Mixed, no. (%) | 0 (0%) |
|  | Fungus, no. (%) | 0 (0%) |
|  | Gram negative, no. (%) | 0 (0%) |
|  | Gram positive, no. (%) | 0 (0%) |
|  | Organism negative^$^, no. (%) | 0 (0%) |
| Drug resistance, no. (%) | | 0 (0%) |

^$^ Organism culture negative or not obtained.

Abbreviations: ALT: alanine transaminase; AST: aspartate transaminase; BUN: blood urea nitrogen; GCS: Glasgow coma score; PaO_2_: partial pressure of oxygen; SBP: systolic blood pressure; WBC: white blood cell

**Supplemental Table 2. Variables before and after multiple imputation**

| **Variable** | **Observed** | **Imputed*** |
| --- | --- | --- |
| Age, median [IQR] | 64 [49 - 74] | 64 [49 - 74] |
| Albumin, g/dL, median [IQR] | 2.0 [1.6- 2.4] | 2.0 [1.6- 2.4] |
| ALT, U/L, median [IQR] | 28 [16 - 55] | 28 [16 -55] |
| AST, U/L, median [IQR] | 43 [24 -93] | 43 [24 -93] |
| Bands, median [IQR] | 1.2 [0.5 -3.1] | 1.1 [0.5 -2.6] |
| Bilirubin, mg/dL, median [IQR] | 0.7 [0.4 -1.3] | 0.7 [0.4 -1.3] |
| BUN, mg/dL, median [IQR] | 10 [6 - 15] | 10 [6 - 15] |
| Chlorine, mmol/L, median [IQR] | 106 [102 - 111] | 106 [101 - 111] |
| Creatinine, mg/dL, median [IQR] | 1.5 [1.0 - 2.3] | 1.5 [1.0 - 2.3] |
| Elixhauser comorbidities, median [IQR] | 1 [0 - 2] | 1 [0 - 2] |
| GCS, median [IQR] | 14 [11 - 15] | 14 [11 - 15] |
| Gender; male, no. (%) | 964 (57%) | 964 (57%) |
| Glucose, mg/dL, median [IQR] | 146 [115 - 198] | 146 [115 - 196] |
| Heart rate, median [IQR] | 130 [115 - 147] | 130 [115 - 147] |
| Hemoglobin, g/dL, median [IQR] | 11 [10 - 12] | 11 [9 - 12] |
| Oxygen saturation, %, median [IQR] | 95 [90 - 97] | 95 [90 - 97] |
| PaO_2_, mmHg, median [IQR] | 76 [62 - 101] | 76 [62 - 101] |
| Prothrombin time, s, median [IQR] | 19 [17 - 22] | 19 [17 - 22] |
| Platelets, ×10^9^/L, median [IQR] | 168 [105 - 240] | 168 [105 - 240] |
| Respiratory rate, /min, median [IQR] | 31 [23 - 40] | 31 [23 - 40] |
| Sodium, mmol/L, median [IQR] | 139 [135 - 143] | 139 [135 - 143] |
| SBP, mmHg, median [IQR] | 80 [70 - 95] | 80 [70 - 95] |
| Temperature, ℃, median [IQR] | 38.6 [37.6 - 39.3] | 38.6 [37.6 - 39.3] |
| WBC Count, ×10^9^/L, median [IQR] | 14 [9 - 20] | 14 [9 - 20] |

*Summary statistics are shown from a random one of 10 datasets imputed

Abbreviations: ALT: alanine transaminase; AST: aspartate transaminase; BUN: blood urea nitrogen; GCS: Glasgow coma score; IQR: interquartile range; PaO_2_: partial pressure of oxygen; SBP: systolic blood pressure; WBC: white blood cell

**Supplemental Table 3. Statistical output from latent class analysis**

|  |  | **statistic** | | | **Class size** | | | | | | |  | |
| --- | --- | --- | --- | --- | --- | --- | --- | --- | --- | --- | --- | --- | --- |
| **Phenotype model** | **Class number** | **BIC** | **Entropy** | **Probability of group membership, %, median [IQR]** | **1** | **2** | **3** | **4** | **5** | **6** | **7** | |  |
| **Host model** | 2 | 137557 | 0.75 | 99.0 [91.4 - 99.9] | 1005 (60%) | 685 (41%) |  |  |  |  |  | |  |
|  | 3 | 136515 | 0.77 | 97.1 [83.7 -99.8] | 420 (25%) | 577 (34%) | 693 (41%) |  |  |  |  | |  |
|  | 4 | 136002 | 0.78 | 95.2 [78.9 - 99.4] | 327 (19%) | 518 (31%) | 532 (32%) | 313 (19%) |  |  |  | |  |
|  | 5 | 135540 | 0.80 | 95.1 [81.1 - 99.5] | 272 (16%) | 390 (23%) | 523 (31%) | 294 (17%) |  |  |  | |  |
|  | 6 | 135330 | 0.81 | 95.1 [79.4 - 99.3] | 231 (14%) | 181 (11%) | 294 (17%) | 261 (15%) | 342 (20%) | 381 (23%) |  | |  |
|  | 7 | 135419 | 0.80 | 92.5 [73.1 - 99.0] | 316 (19%) | 218 (13%) | 287 (17%) | 344 (20%) | 167 (10%) | 274 (16%) | 84 (5.0%) | |  |
|  |  |  |  |  |  |  |  |  |  |  |  | |  |
| **Host-pathogen model** | 2 | 149146 | 0.77 | 99.4 [93.4 - 100] | 1125 (67%) | 565 (33%) |  |  |  |  |  | |  |
|  | 3 | 148188 | 0.77 | 97.4 [83.3 - 99.8] | 411 (24%) | 604 (36%) | 675 (40%) |  |  |  |  | |  |
|  | 4 | 147606 | 0.79 | 97.1 [81.7 - 99.8] | 358 (21%) | 519 (31%) | 374 (22%) | 439 (26%) |  |  |  | |  |
|  | 5 | 147317 | 0.81 | 96.3 [82.2 - 99.7] | 301 (18%) | 411 (24%) | 135 (8.0%) | 384 (23%) | 459 (27%) |  |  | |  |
|  | 6 | 147029 | 0.82 | 96.1 [80.0 - 99.6] | 199 (12%) | 361 (21%) | 418 (25%) | 217 (13%) | 259 (15%) | 236 (14%) |  | |  |
|  | 7 | 146939 | 0.83 | 95.7 [77.9 - 99.5] | 101 (6.0%) | 212 (13%) | 203 (12%) | 310 (18%) | 304 (18%) | 345 (20%) | 215 (13%) | |  |

Abbreviations: BIC: Bayesian information criteria; IQR: interquartile range

**Supplemental Table 4. Microbiological characteristics and outcomes by host phenotypes**

| **Variable** | | **All patients** | **α-type** | **β-type** | **γ-type** | **δ-type** | **P-value*** |
| --- | --- | --- | --- | --- | --- | --- | --- |
| No. | | 1,690 | 327 (19%) | 518 (31%) | 532 (32%) | 313 (19%) |  |
| **Microbiological variables** | |  |  |  |  |  |  |
| Infection site | |  |  |  |  |  | <0.01 |
|  | Bloodstream, no. (%) | 87 (5.2%) | 13 (4.0%) | 17 (3.3%) | 27 (5.1%) | 30 (10%) |  |
|  | Central nervous system, no. (%) | 39 (2.3%) | 11 (3.4%) | 11 (2.1%) | 6 (1.1%) | 11 (3.5%) |  |
|  | Genitourinary, no. (%) | 179 (11%) | 22 (6.7%) | 51 (10%) | 68 (13%) | 38 (12%) |  |
|  | Abdominal, no. (%) | 337 (19%) | 55 (17%) | 102 (20%) | 120 (23%) | 60 (19%) |  |
|  | Lung, no. (%) | 906 (54%) | 205 (63%) | 293 (57%) | 268 (50%) | 140 (45%) |  |
|  | Others, no. (%) | 142 (8.4%) | 21 (6.4%) | 44 (9%) | 43 (8.1%) | 34 (11%) |  |
| Type | |  |  |  |  |  | <0.01 |
|  | Mixed, no. (%) | 596 (35%) | 121 (37%) | 188 (36%) | 183 (34%) | 104 (33%) |  |
|  | Fungus, no. (%) | 85 (5.0%) | 19 (5.8%) | 37 (7.1%) | 18 (3.4%) | 11 (3.5%) |  |
|  | Gram negative, no. (%) | 264 (16%) | 33 (10%) | 78 (15%) | 92 (17%) | 61 (20%) |  |
|  | Gram positive, no. (%) | 367 (22%) | 72 (22%) | 87 (17%) | 130 (24%) | 78 (25%) |  |
|  | Organism negative^$^, no. (%) | 378 (22%) | 82 (25%) | 128 (25%) | 109 (21%) | 59 (19%) |  |
| Drug resistance, no. (%) | | 499 (30%) | 249 (41%) | 133 (32%) | 116 (28%) | 1 (0.4%) | <0.01 |
| **Outcomes** | |  |  |  |  |  |  |
|  | 28-day mortality, no. (%) | 469 (28%) | 29 (9%) | 157 (30%) | 152 (29%) | 131 (42%) | <0.01 |
|  | 90-day mortality, no. (%) | 593 (35%) | 43 (13%) | 210 (41%) | 188 (35%) | 152 (49%) | <0.01 |
|  | 180-day mortality, no. (%) | 638 (38%) | 51 (16%) | 233 (45%) | 198 (37%) | 156 (50%) | <0.01 |

^$^ Organism culture negative or not obtained.

*Kruskal-Wallis, or chi-square P-value as appropriate comparing across all four phenotypes

Abbreviations: ALT: alanine transaminase; AST: aspartate transaminase; BUN: blood urea nitrogen; GCS: Glasgow coma score; IQR, interquartile range; PaO_2_: partial pressure of oxygen; SBP: systolic blood pressure; WBC: white blood cell

**Supplemental Table 5. Metrics of model fit between host and clinical and host-pathogen models**

|  | | **Host model** | **Host-pathogen model** |
| --- | --- | --- | --- |
| BIC | | 136002 | 147606 |
| Entropy | | 0.78 | 0.79 |
| Probability of Membership >90%, N (%) | |  |  |
|  | α-type | 225/327 (68.8%) | 246/358 (68.7%) |
|  | β-type | 313/518 (60.4%) | 330/519 (63.6%) |
|  | γ-type | 261/532 (49.1%) | 191/374 (51.1%) |
|  | δ-type | 235/313 (75.1%) | 339/439 (77.2%) |
|  | Total | 1034/1690 (61.2%) | 1106/1690 (65.4%) |
| Probability of total, %, median [IQR] | | 95.2 [78.9 – 99.4] | 97.1 [81.7 - 99.8] |

Abbreviations: BIC: Bayesian information criteria; IQR: interquartile range

**Supplemental Table 6. Comparison of the probability between membership changed and not changed patients when microbiological variables added**

| **Phenotype** | **Number (%)** | **Probability of membership among those who changed phenotype when microbiological variables added** | **Probability of membership among those who did not change phenotype when microbiological variables added** | **P-value** |
| --- | --- | --- | --- | --- |
| α-type | 68/327 (20.8%) | 78.1 [62.9 - 94.2] | 98.5 [93.4 - 99.9] | <0.01 |
| β-type | 232/518 (44.8%) | 92.5 [74.7 - 99.0] | 96.0 [82.8 - 99.4] | <0.01 |
| γ-type | 427/532 (80.3%) | 91.3 [76.0 - 98.0] | 81.4 [68.3 - 93.8] | <0.01 |
| δ-type | 45/313 (14.4%) | 80.3 [63.4 - 95.6] | 99.9 [94.8 - 100] | <0.01 |
| Total | 772/1690 (45.7%) | 90.3 [73.4 - 98.0] | 97.8 [86.8 - 99.9] | <0.01 |

Note: the probability in this table was the membership probability of host phenotypes (%, median [IQR]).

**Supplemental Table 7.** **Characteristics of variables by host-pathogen phenotypes**

| **Characteristic** | **All patients** | **α-type** | **β-type** | **γ-type** | **δ-type** | **P-value*** |
| --- | --- | --- | --- | --- | --- | --- |
| No. of patients (%) | 1,690 | 358 (21%) | 519 (31%) | 374 (22%) | 439 (26%) |  |
| Age, median [IQR], years | 64 [49 - 74] | 44 [34 - 53] | 69 [58 - 77] | 70 [61 - 77] | 61 [48 - 74] | <0.01 |
| Gender, no. (%) |  |  |  |  |  |  |
| Male | 964 (57%) | 213 (60%) | 277 (53%) | 230 (62%) | 244 (56%) | 0.07 |
| Female | 726 (43%) | 145 (40%) | 242 (47%) | 144 (38%) | 195 (44%) |  |
| Elixhauser Comorbidities, median [IQR] | 1 [0 - 2] | 0 [0 - 1] | 1 [1 - 2] | 2 [1 - 3] | 1 [0 - 2] | <0.01 |
|  |  |  |  |  |  |  |
| **Inflammation** |  |  |  |  |  |  |
| Premature neutrophil count (bands), median [IQR], % | 1.1 [0.5 - 2.6] | 0.9 [0.4 - 1.5] | 1.1 [0.5 - 2.6] | 1.2 [0.5 - 2.6] | 1.3 [0.5 - 3.5] | <0.01 |
| Temperature, median [IQR], °C | 38.6 [37.6 - 39.3] | 39.0 [38.5 - 39.5] | 38.2 [36.0 - 39.0] | 38.3 [37.2 - 39.1] | 38.8 [37.7 - 39.6] | <0.01 |
| White blood cell count, median [IQR], ×10^9^/L | 14 [9 - 20] | 13 [8 - 18] | 13 [8 - 19] | 16 [12 - 21] | 14 [6 - 23] | <0.01 |
|  |  |  |  |  |  |  |
| **Pulmonary** |  |  |  |  |  |  |
| Oxygen saturation, median [IQR], % | 95 [90 - 97] | 94 [89 - 96] | 97 [94 - 98] | 92 [85 - 95] | 94 [89 - 97] | <0.01 |
| Partial pressure of oxygen, arterial, median [IQR], mmHg | 76 [62 - 101] | 70 [57 - 85] | 92 [73 - 138] | 64 [53 - 77] | 77 [62 - 105] | <0.01 |
| Respiratory rate, median [IQR], breaths/min | 31 [23 - 40] | 35 [25 - 44] | 25 [15 - 32] | 32 [26 - 40] | 33 [26 - 40] | <0.01 |
|  |  |  |  |  |  |  |
| **Cardiovascular or Hemodynamic** |  |  |  |  |  |  |
| Heart rate, median [IQR], beats/min | 130 [115 - 147] | 136 [123 - 148] | 126 [110 - 144] | 127 [112 - 145] | 136 [120 - 150] | <0.01 |
| Systolic blood pressure, median [IQR], mmHg | 80 [70 - 95] | 90 [78 - 106] | 80 [70 - 92] | 83 [69 - 103] | 75 [64 - 85] | <0.01 |
|  |  |  |  |  |  |  |
| **Renal** |  |  |  |  |  |  |
| Blood urea nitrogen, median [IQR], mg/dL | 10 [6 - 15] | 5 [4 - 8] | 10 [7 - 15] | 11 [8 - 16] | 14 [10 - 19] | <0.01 |
| Creatinine, median [IQR], mg/dL | 1.5 [1.0 - 2.3] | 1.0 [0.7 - 1.3] | 1.4 [1.0 - 2.3] | 1.4 [1.0 - 2.3] | 2.3 [1.6 - 3.4] | <0.01 |
|  |  |  |  |  |  |  |
| **Hepatic** |  |  |  |  |  |  |
| Alanine transaminase, median [IQR], U/L | 28 [16 - 55] | 28 [16 - 46] | 19 [12 - 29] | 23.5 [16 - 39] | 93 [40 - 236] | <0.01 |
| Aspartate transaminase, median [IQR], U/L | 43 [24 - 93] | 43 [24 - 80] | 29 [21 - 43] | 36 [22 - 55] | 159 [80 - 407] | <0.01 |
| Bilirubin, median [IQR], mg/dL | 0.7 [0.4 - 1.3] | 0.8 [0.5 - 1.3] | 0.6 [0.4 - 1.1] | 0.6 [0.3 - 0.9] | 1.0 [0.6 - 2.1] | <0.01 |
|  |  |  |  |  |  |  |
| **Hematologic** |  |  |  |  |  |  |
| Hemoglobin, median [IQR], g/dL | 11 [9 - 12] | 11 [10 - 12] | 10 [9 - 11] | 11 [10 - 12] | 11 [10 - 12] | <0.01 |
| Platelets, median [IQR], ×10^9^/L | 168 [105 - 240] | 180.5 [115 - 247] | 175 [116 - 252] | 205 [157 - 281] | 109 [64 - 178] | <0.01 |
| Prothrombin time, median [IQR], secs | 19 [17 - 22] | 18 [16 - 20] | 19 [17 - 23] | 17 [15 - 19] | 22 [18 - 27] | <0.01 |
|  |  |  |  |  |  |  |
| **Other** |  |  |  |  |  |  |
| Albumin, median [IQR], g/dL | 2.0 [1.6 - 2.4] | 2.1 [1.7 - 2.5] | 1.8 [1.4 - 2.2] | 2.3 [1.9 - 2.7] | 1.9 [1.6 - 2.4] | <0.01 |
| Chloride, median [IQR], mEq/L | 106 [101 - 111] | 106 [102 - 110] | 110 [105 - 115] | 102 [98 - 106] | 105 [101 - 110] | <0.01 |
| Glasgow Coma Scale score, median [IQR] | 14 [11 - 15] | 15 [13 - 15] | 14 [10 - 15] | 14 [11 - 15] | 14 [11 - 15] | <0.01 |
| Glucose, median [IQR], mg/dL | 146 [115 - 196] | 133 [111 - 162] | 147 [117 - 198] | 166 [127 - 227] | 146 [106 - 204] | <0.01 |
| Sodium, median [IQR], mEp/L | 139 [135 - 143] | 139 [135 - 142] | 141 [137 - 145] | 137 [133 - 140] | 138 [135 - 143] | <0.01 |
|  |  |  |  |  |  |  |
| **Outcomes** |  |  |  |  |  |  |
| 28-day mortality, no. (%) | 469 (28%) | 45 (13%) | 132 (25%) | 110 (29%) | 181 (42%) | <0.01 |
| 90-day mortality, no. (%) | 593 (35%) | 57 (16%) | 186 (36%) | 140 (37%) | 210 (48%) | <0.01 |
| 180-day mortality, no. (%) | 638 (38%) | 63 (18%) | 206 (40%) | 153 (41%) | 216 (49%) | <0.01 |

*Kruskal-Wallis used for continuous and or chi-square for categorical comparisons, across four phenotypes

Abbreviations: IQR, interquartile range.

**Supplemental Table 8: Characteristics of Microbiological variable by phenotypes in host-pathogen model**

| **Microbiological variables** | | **All patients** | **α-type** | **β-type** | **γ-type** | **δ-type** | **P-value** |
| --- | --- | --- | --- | --- | --- | --- | --- |
| Infection site | |  |  |  |  |  | <0.01 |
|  | Bloodstream, no. (%) | 87 (5.2%) | 19 (5.3%) | 16 (3.1%) | 2 (0.5%) | 50 (11.4%) |  |
|  | Central nervous system, no. (%) | 39 (2.3%) | 10 (2.8%) | 16 (3.1%) | 4 (1.1%) | 9 (2.1%) |  |
|  | Genitourinary, no. (%) | 179 (10.6%) | 16 (4.5%) | 58 (11.2%) | 38 (10.2%) | 67 (15.3%) |  |
|  | Abdominal, no. (%) | 337 (19.4%) | 44 (12.3%) | 208 (40.1%) | 8 (2.1%) | 77 (17.5%) |  |
|  | Lung, no. (%) | 906 (53.6%) | 243 (67.9%) | 183 (35.3%) | 291 (77.8%) | 189 (43.1%) |  |
|  | Others, no. (%) | 142 (8.4%) | 26 (7.3%) | 38 (7.3%) | 31 (8.3%) | 47 (10.7%) |  |
| Type | |  |  |  |  |  | <0.01 |
|  | Mixed, no. (%) | 596 (35.3%) | 131 (36.6%) | 230 (44.3%) | 96 (25.7%) | 139 (31.7%) |  |
|  | Fungus, no. (%) | 85 (5.0%) | 22 (6.2%) | 28 (5.4%) | 24 (6.4%) | 11 (2.5%) |  |
|  | Gram negative, no. (%) | 264 (15.6%) | 23 (6.4%) | 84 (16.2%) | 58 (15.5%) | 99 (22.6%) |  |
|  | Gram positive, no. (%) | 367 (21.7%) | 99 (27.7%) | 87 (16.8%) | 67 (17.9%) | 114 (26.0%) |  |
|  | Organism negative, no. (%) | 378 (22.4%) | 83 (23.2%) | 90 (17.3%) | 129 (34.5%) | 76 (17.3%) |  |
| Drug resistance, no. (%) | | 499 (29.5%) | 116 (32.4%) | 198 (38.2%) | 64 (17.1%) | 121 (27.6%) | <0.01 |

**Supplemental Table 9. Biomarkers by host phenotypes**

| **Biomarkers** | **All patients** | **α-type** | **β-type** | **γ-type** | **δ-type** | **P-value** |
| --- | --- | --- | --- | --- | --- | --- |
| Antithrombin, median [IQR], mg/mL | 0.59 [0.44 - 0.75] | 0.67 [0.54 - 0.81] | 0.67 [0.54 - 0.82] | 0.53 [0.4 - 0.66] | 0.48 [0.33 - 0.65] | <0.01 |
| D-Dimer, median [IQR], μg/mL | 4.2 [2.2 - 8.3] | 3.4 [1.8 - 6.5] | 3.2 [1.8 - 6.6] | 4.7 [2.7 - 8.9] | 6.9 [3.5 - 15.1] | <0.01 |
| Factor V, median [IQR], % of normal | 84 [62 - 105] | 102 [83 - 122.5] | 99 [83 - 111] | 64.5 [56 - 89] | 46 [44 - 67] | <0.01 |
| IL-1b, median [IQR] , pg/mL | 10 [10 - 10] | 11 [10 - 10] | 12 [10 - 10] | 13 [10 - 10] | 14 [10 - 10] | 0.45 |
| IL-6, median [IQR], pg/mL | 492 [1434 - 2574] | 277 [107 - 873] | 255 [93 - 861] | 1181 [279 - 6019] | 1228 [253 - 10565] | <0.01 |
| IL-8, median [IQR], pg/mL | 50 [50 - 227] | 50 [50 - 50] | 50 [50 - 50] | 117 [50 - 422] | 179.5 [50 - 839.5] | <0.01 |
| IL-10, median [IQR], pg/mL | 10 [10 - 41] | 10 [10 - 25] | 10 [10 - 26] | 10 [10 - 56] | 39 [10 - 91] | <0.01 |
| PAI-1, median [IQR], AU/mL | 34 [20 - 64] | 24 [15 - 37] | 25 [17 - 40] | 41 [25 - 81] | 66 [33 - 89] | <0.01 |
| Plasminogen activity, median [IQR], % | 61 [49 - 75] | 68 [56 - 81] | 69 [56 - 82] | 55 [43 - 63] | 50 [38 - 65] | <0.01 |
| Protein C Activity, median [IQR], % | 48 [31 - 65] | 56 [41 - 74] | 58 [43 - 80] | 40 [28 - 55] | 34 [20 - 50] | <0.01 |
| Protein S Activity, median [IQR], % | 36 [22 - 57] | 50 [31 - 66] | 46 [26 - 63] | 32 [20 - 52] | 29 [15 - 47] | <0.01 |
| Prothrombin fragment 1-2, median [IQR], nmol/L | 1.8 [1.1 - 2.6] | 1.7 [1.0 - 2.3] | 1.5 [1.1 - 2.4] | 1.8 [1.1 - 2.6] | 2.2 [1.4 - 3.5] | 0.01 |
| TAT complex, median [IQR], μg/dL | 11 [7.4 - 19.7] | 9.8 [6.5 - 18.0] | 10.0 [6.4 - 16.7] | 12.0 [8.0 - 18.0] | 19.6 [11.6 - 39.5] | <0.01 |
| TNF-α, median [IQR], pg/mL | 21 [10 - 52] | 10 [10 - 26] | 10 [10 - 36] | 33 [10 - 66] | 37 [10 - 96] | <0.01 |

Abbreviations: IL: interleukin; IQR: interquartile range; PAI: plasminogen activator inhibitor, TAT: thrombin-antithrombin; TNF: tumor necrosis factor

**Supplemental Table 10. Biomarkers by host-pathogen phenotypes**

| **Biomarkers** | **All patients** | **α-type** | **β-type** | **γ-type** | **δ-type** | **P-value** |
| --- | --- | --- | --- | --- | --- | --- |
| Antithrombin, median [IQR], mg/mL | 0.59 [0.44 - 0.75] | 0.64 [0.52 - 0.76] | 0.54 [0.42 - 0.70] | 0.72 [0.59 - 0.86] | 0.49 [0.35 - 0.64] | <0.01 |
| D-Dimer, median [IQR], μg/mL | 4.2 [2.2 - 8.3] | 3.5 [2.0 - 6.4] | 4.2 [2.3 - 7.5] | 3.0 [1.6 - 6.0] | 7.1 [3.6 - 13.9] | <0.01 |
| Factor V, median [IQR], % of normal | 84 [62 - 105] | 95 [76 - 120] | 89 [60 - 99] | 91 [83 - 133] | 56 [46 - 67] | 0.02 |
| IL-1b, median [IQR] , pg/mL | 10 [10 - 10] | 10 [10 - 10] | 10 [10 - 10] | 10 [10 - 10] | 10 [10 - 10] | 0.42 |
| IL-6, median [IQR], pg/mL | 492 [144 - 2574] | 348 [135 - 1346] | 463 [150 - 1858] | 276 [95 - 1326] | 1561 [289 - 13004] | <0.01 |
| IL-8, median [IQR], pg/mL | 50 [50 - 227] | 50 [50 - 50] | 50 [50 - 199] | 50 [50 - 122] | 168 [50 - 839] | <0.01 |
| IL-10, median [IQR], pg/mL | 10 [10 - 41] | 10 [10 - 27] | 10 [10 - 30] | 10 [10 - 27] | 39 [10 - 73] | <0.01 |
| PAI-1, median [IQR], AU/mL | 34 [20 - 64] | 27 [17 - 39] | 35 [19 - 54] | 24 [18 - 38] | 73 [34 - 90] | <0.01 |
| Plasminogen activity, median [IQR], % | 61 [49 - 75] | 64 [52 - 77] | 57 [44 - 75] | 72 [59 - 85] | 50 [41 - 64] | <0.01 |
| Protein C Activity, median [IQR], % | 48 [31 - 65] | 51 [37 - 67] | 46 [31 - 65] | 59 [46 - 79] | 36 [23 - 50] | <0.01 |
| Protein S Activity, median [IQR], % | 36 [22 - 57] | 47 [29 - 61] | 30 [20 - 52] | 53 [32 - 70] | 30 [17 - 48] | <0.01 |
| Prothrombin fragment 1-2, median [IQR], nmol/L | 1.8 [1.1 - 2.6] | 1.5 [1.0 - 2.4] | 1.7 [1.1 - 2.4] | 1.5 [1.2 - 2.4] | 2.1 [1.4 - 3.5] | <0.01 |
| TAT complex, median [IQR], μg/dL | 11.0 [7.4 - 19.7] | 9.5 [6.6 - 17.5] | 10.4 [7.2 - 16.6] | 10.1 [6.5 - 19.7] | 16.1 [10.0 - 28.5] | <0.01 |
| TNF-α, median [IQR], pg/mL | 21 [10 - 52] | 10 [10 - 29] | 10 [10 - 35] | 21 [10 - 51] | 57 [23 - 111] | <0.01 |

Abbreviations: IL: interleukin; IQR: interquartile range; PAI: plasminogen activator inhibitor, TAT: thrombin-antithrombin; TNF: tumor necrosis factor

**Supplemental Table 11. Statistical output from latent class analysis in sensitivity analysis excluding variables with high missingness (Hemoglobin, premature white cells (%bands))**

|  |  | **statistic** | | | **Class size** | | | | | |  |  |
| --- | --- | --- | --- | --- | --- | --- | --- | --- | --- | --- | --- | --- |
| **Phenotype model** | **Class number** | **BIC** | **Entropy** | **Probability of group membership, %, median [IQR]** | **1** | **2** | **3** | **4** | **5** | **6** | **7** | |
| **Host model** | 2 | 124757 | 0.77 | 99.3 [93.3 - 99.9] | 1164 (69%) | 526 (31%) |  |  |  |  |  | |
|  | 3 | 123781 | 0.76 | 97.2 [83.5 - 99.8] | 499 (30%) | 484 (29%) | 707 (42%) |  |  |  |  | |
|  | 4 | 123244 | 0.79 | 95.7 [79.3 - 99.7] | 331 (20%) | 635 (38%) | 362 (21%) | 362 (21%) |  |  |  | |
|  | 5 | 122890 | 0.79 | 95.1 [78.3 - 99.4] | 464 (28%) | 492 (29%) | 159 (9.4%) | 277 (16%) | 298 (18%) |  |  | |
|  | 6 | 122532 | 0.81 | 94.8 [79.5 - 99.3] | 370 (22%) | 177 (11%) | 292 (17%) | 414 (25%) | 236 (14%) | 201 (12%) |  | |
|  | 7 | 122469 | 0.81 | 92.9 [76.4 - 99.0] | 338 (20%) | 198 (12%) | 384 (23%) | 228 (14%) | 309 (18%) | 142 (8.4%) | 91 (5.4%) | |
|  |  |  |  |  |  |  |  |  |  |  |  | |
| **Host-pathogen model** | 2 | 114585 | 0.77 | 99.3 [93.1 - 99.9] | 562 (33%) | 1128 (67%) |  |  |  |  |  | |
|  | 3 | 113988 | 0.77 | 97.3 [83.6 - 99.8] | 706 (42%) | 419 (25%) | 565 (33%) |  |  |  |  | |
|  | 4 | 113585 | 0.78 | 95.9 [81.9 - 99.7] | 464 (28%) | 465 (28%) | 360 (21%) | 401 (24%) |  |  |  | |
|  | 5 | 113438 | 0.81 | 95.8 [81.6 - 99.6] | 438 (26%) | 405 (24%) | 255 (15%) | 400 (24%) | 192 (11%) |  |  | |
|  | 6 | 113430 | 0.82 | 95.2 [79.6 - 99.5] | 107 (6.3%) | 371 (22%) | 383 (23%) | 215 (13%) | 232 (14%) | 382 (23%) |  | |
|  | 7 | 113394 | 0.82 | 94.5 [78.9 - 99.5] | 227 (13%) | 334 (20%) | 313 (19%) | 172 (10%) | 225 (13%) | 117 (6.9%) | 302 (18%) | |

Abbreviations: BIC: Bayesian information criteria; IQR: interquartile range

**Supplemental Table 12. Metrics of model fit in 5-class model between host and host-pathogen phenotypes**

|  | | **Host model** | **Host-pathogen model** |
| --- | --- | --- | --- |
| BIC | | 135540 | 147317 |
| Entropy | | 0.80 | 0.81 |
| Probability of Membership >90%, N (%) | |  |  |
|  | Type 1 | 209/294 (71%) | 205/301 (68%) |
|  | Type 2 | 312/523 (60%) | 77/135 (57%) |
|  | Type 3 | 220/390 (56%) | 221/384 (58%) |
|  | Type 4 | 207/272 (76%) | 327/411 (80%) |
|  | Type 5 | 107/211 (51%) | 271/459 (59%) |
|  | Total | 1055/1690 (62%) | 1101/1690 (65%) |
| Probability of total, %, median [IQR] | | 95.2 [81.0 - 99.5] | 96.3 [82.2 - 99.7] |

Abbreviations: BIC: Bayesian information criteria; IQR: interquartile range

**Supplemental Table 13. Comparison of microbiological variables in type 2 and type 3 of 5-class model between host and host-pathogen phenotypes**

| **Variable** | | **Host type 2** | **Host-pathogen type 2** |  | **Host type 3** | **Host-pathogen type 3** |
| --- | --- | --- | --- | --- | --- | --- |
| **No.** | | 523 (31%) | 135 (8.0%) |  | 390 (23%) | 384 (23%) |
| **Microbiological variable** | |  |  |  |  |  |
| Infection site | |  |  |  |  |  |
|  | Bloodstream, no. (%) | 16 (3.1%) | 13 (10%) |  | 28 (7.2%) | 2 (0.5%) |
|  | Central nervous system, no. (%) | 10 (1.9%) | 3 (2.2%) |  | 5 (1.3%) | 14 (3.7%) |
|  | Genitourinary, no. (%) | 55 (11%) | 16 (12%) |  | 53 (14%) | 49 (13%) |
|  | Abdominal, no. (%) | 143 (27%) | 21 (16%) |  | 78 (20%) | 187 (49%) |
|  | Lung, no. (%) | 250 (48%) | 69 (51%) |  | 192 (49%) | 110 (29%) |
|  | Others, no. (%) | 49 (9.4%) | 13 (10%) |  | 34 (8.7%) | 22 (5.7%) |
| Type | |  |  |  |  |  |
|  | Mixed, no. (%) | 201 (38%) | 49 (36%) |  | 129 (33%) | 187 (49%) |
|  | Fungus, no. (%) | 31 (5.9%) | 11 (8.2%) |  | 13 (3.3%) | 20 (5.2%) |
|  | Gram negative, no. (%) | 75 (14%) | 14 (10%) |  | 72 (19%) | 63 (16%) |
|  | Gram positive, no. (%) | 100 (19%) | 30 (22%) |  | 100 (26%) | 52 (14%) |
|  | Organism negative, no. (%) | 116 (22%) | 31 (23%) |  | 76 (20%) | 62 (16%) |
| Drug resistance, no. (%) | | 165 (32%) | 46 (34%) |  | 119 (31%) | 154 (40%) |
